# Sevoflurane anesthesia ameliorates LPS-induced acute lung injury (ALI) by modulating a novel LncRNA LINC00839/miR-223/NLRP3 axis

**DOI:** 10.1186/s12890-022-01957-5

**Published:** 2022-04-26

**Authors:** Zhiling Fu, Xiuying Wu, Fushuang Zheng, Yan Zhang

**Affiliations:** 1 Department of Anesthesiology, Shengjing Hospital of China Medical University, No. 36 Sanhao Street, Shenyang, 110004 Liaoning China; 2 Department of Thoracic Surgery, Shengjing Hospital of China Medical University, No. 36 Sanhao Street, Shenyang, 110004 Liaoning China

**Keywords:** Acute lung injury, Sevoflurane, LINC00839, miR-223, NLRP3, Pyroptosis

## Abstract

**Background:**

Sevoflurane is considered as a lung-protective factor in acute lung injury (ALI), but the underlying molecular mechanism remains largely unknown. The present study identified for the first time that sevoflurane ameliorated lipopolysaccharide (LPS)-induced ALI through regulating a novel long non-coding RNA LINC00839, and uncovered its regulatory mechanism.

**Methods:**

LPS-induced ALI models were established in mice or mouse pulmonary microvascular endothelial cells (MPVECs), and they were administered with sevoflurane. Real-Time quantitative PCR, western blot and bioinformatics analysis were performed to screen the aberrantly expressed long non-coding RNA and the downstream molecules in sevoflurane-treated ALI models, and their roles in the protection effect of sevoflurane were verified by functional recovery experiments.

**Results:**

Sevoflurane relieved LPS-induced lung injury, cell pyroptosis and inflammation in vitro and in vivo. LINC00839 was significantly suppressed by sevoflurane, and overexpression of LINC00839 abrogated the protective effects of sevoflurane on LPS-treated MPVECs. Mechanismly, LINC00839 positively regulated NOD-like receptor protein 3 (NLRP3) via sequestering miR-223. MiR-223 inhibitor reversed the inhibitory effects of LINC00839 knockdown on NLRP3-mediated pyroptosis in LPS-treated MPVECs. Furthermore, both miR-223 ablation and NLRP3 overexpression abrogated the protective effects of sevoflurane on LPS-treated MPVECs.

**Conclusion:**

In general, our work illustrates that sevoflurane regulates the LINC00839/miR-223/NLRP3 axis to ameliorate LPS-induced ALI, which might provide a novel promising candidate for the prevention of ALI.

## Introduction

Acute lung injury (ALI) is a serious complication of multiple diseases, with high incidence and mortality in clinic [1, 2]. At present, the main strategies for ALI treatment are to control the primary disease, and curb the subsequent systemic inflammatory response. Actually, there is short of good therapeutic drugs, and the treatment of ALI poses a formidable challenge due to the complexity of the disease. In recent years, new treatment drugs or methods have been appearing in clinic, mainly to reduce the inflammatory response, correct hypoxia, or promote the absorption of lung fluid [3]. However, their in-depth mechanism is largely unknown. Therefore, it is of great significance to explore the molecular mechanism of those drugs and find the effective therapeutic targets for ALI.

Volatile anesthetics have organ-protective effects in a variety of pathological conditions [4]. Sevoflurane is an inhaled anesthetic widely used in general anesthesia. For example, evidences showed that sevoflurane can maintain cell viability in neuron myocardial tissue [5–7]. Recently, sevoflurane has been reported to have a lung-protective effect in ALI [8–11], but the mechanism has not been fully clarified. A study from Wang et al. [11] indicated that sevoflurane alleviates LPS-induced ALI through suppressing the nuclear factor kappa-B pathway-mediated inflammatory response. Another study by Wang et al. [10] pointed that sevoflurane can ameliorated allergic airway inflammation by inhibiting the expression of NOD-like receptor protein 3 (NLRP3). Similarly, they only mentioned the inflammation phenotype of ALI in previous related studies. As is well-known, hyperinflammatory response in lung is one of the mechanisms of ALI development, and NLRP3 plays a critical role in the progression of inflammation [12–14]. Significantly, NLRP3 inflammasomes not only promotes the release of a variety of inflammatory factors, but also induces cell pyroptosis, a mode of cell death with inflammatory characteristics [15–18]. Nevertheless, it is unclear about the association between the protection of sevoflurane on lung tissues with NLRP3-mediated pyroptosis.

Long non-coding RNAs (LncRNAs) are a class of RNAs that do not encode proteins, with a length of 200 nucleotides at least. The role of LncRNAs in epigenetic regulation has attracted extensive attention in recent 10 years. LncRNAs participates in almost all physiological and pathological processes by directly or indirectly regulating protein expression, including inflammation or pyroptosis in ALI [19, 20]. A study by Zhou et al. [21] demonstrated depression of LncRNA NEAT1 antagonizes LPS-evoked acute injury and inflammatory response. Another study by Qiu et al. [22] suggested that LncRNA TUG1 alleviates sepsis-induced ALI. Up until now, plenty of LncRNAs have been identified and validated in the human genome. LINC00839, a novel LncRNA, has been reported as an oncogene in several cancers, such as Osteosarcoma [23], Neuroblastoma [24], hepatocellular carcinoma [25]. In the present study, we firstly revealed its downregulation by sevoflurane in ALI models in vivo and in vitro. Its role in ALI has not been studied before.

According to the classic LncRNAs-miRNAs-mRNA competing endogenous RNA (ceRNA) network mechanisms, LINC00839 might act as a sponge for microRNA (miRNA) to elevate the expression of mRNA. Our bioinformatics analysis showed that LINC00839 were capable of binding to miR-223. Notably, miR-223 is considered a key regulator in the body anti-inflammation response [26, 27]. It can inhibit lung inflammation in ALI by targeting NLRP3 [17, 28, 29]. Thereupon, we hypothesized that sevoflurane might protect lung tissues through downregulating LINC00839/miR-223/NLRP3 axis in ALI. In this study, we constructed ALI models through LPS inducement in vivo and in vitro, and treated them with sevoflurane to investigate the role of the LINC00839/miR-223/NLRP3 axis in lung protection of sevoflurane. The present work aimed to reveal the potential molecular mechanism of sevoflurane on lung protection, and provide a promising intervention method for lung injury.

## Materials and methods

### Animal treatment

Male C57BL/6 J mice (n = 12, aged 6–8 weeks, weighing 20–25 g), were purchased from Liaoning University of traditional Chinese Medicine (Production license: SCXK(Liao)2019-0001). All mice were maintained in a sterile climate control area with a humidity of 40–60% at about 25 °C, in a 12 h light/12 h dark cycle. And they were free for sufficient water and food. The procedures for care and use of animals were approved by the animal experiment ethics committee of Liaoning University of Traditional Chinese Medicine.

The treatment methods of LPS and sevoflurane referred to previous studies [11, 30]. Mice were randomly divided into 4 groups: control group (Con), sevoflurane group (Se), LPS group (LPS) and LPS + sevoflurane group (LPS + Se). Mice in LPS and LPS + Se were infused with 3 mg/kg LPS through trachea. 2 h later, 3% sevoflurane were infused through trachea to the mice in Se and LPS + Se for 4 h. Then the mice were sacrificed after 2 days.

### Cell culture and treatments

Mouse pulmonary microvascular endothelial cells (MPVECs) were purchased from ATCC cell resource center in China. MPVECs were cultured in the DMEM/F12 culture medium containing 10% fetal bovine serum (Thermo Fisher Scientific, Shanghai, China) and 1% double antibiotics (Penicillin–Streptomycin Solution, Beyotime, Shanghai, China). Then they were kept in an incubator supplied with 5% CO_2_ at 37 °C.

MPVECs were first divided into 4 groups, the treatment methods of LPS and sevoflurane referred to previous studies [31]: Con, cells were given no treatment. Se, cells were exposed to 3% sevoflurane for 1 h. LPS, cells were subjected to 1 μg/mL LPS treatment. LPS + Se, cells were exposed to sevoflurane 3 h after LPS treatment.

### Cell transfection

MPVECs (1 × 10^5^ per milliliter) were transfected with 20 μmol/L pcDNA-LINC00839, siRNA-LINC00839 or pcDNA-NLRP3, 200 nmol/L miR-223 inhibitor or miR-223 inhibitor, correspondingly. The transfection was performed using Lipofectamine 2000 (Invitrogen, CA, USA). 24 h after transfection, cells were treated with 1 μg/mL LPS treatment for another 24 h, and exposed to 3% sevoflurane for 1 h. In this section, pcDNA-LINC00839, pcDNA-NLRP3, siRNA-LINC00839 and siRNA-LINC00839 were synthesized in HanBio company (Shanghai, China). NC mimic, miR-223 inhibitor and miR-223 mimic were purchased from Thermo Fisher company (Shanghai, China).

### Hematoxylin & Eosin(H&E) staining assay

Mice were euthanized, and the left lung tissues were made into paraffin sections. Histopathological changes were observed by H&E staining assay as previously reported [32]. Briefly, the paraffin sections were dewaxed with xylene, rewatered with gradient ethanol, and stained with hematoxylin and eosin successively. Then they were dehydrated and dried, sealed with neutral gum, and observed under the microscope (CKX53, Olympus, Tokyo, Japan). The histopathological score was assessed by double-blind method as previous described [33].

### The wet/dry weight ratio

The right lungs were excised and weighed after mice were sacrificed. Subsequently, the lungs were placed in an 80 °C incubator for 48 h, and weighed again to obtain the dry weight. The wet/dry weight ratio was calculated to evaluate tissue edema.

### The capillary permeability assessment

Lung permeability was assessed by Evans blue staining as previously reported [34]. In short, mice were firstly administered 2% Evans blue (Solarbio, Beijing, China) through the tail vein 1 h before euthanasia. Then, Evans blue dye was extracted from the lung by formamide (Solarbio, Beijing, China) at 60 °C for 18 h. The absorbance of the supernatant at 620 nm was measured on a multimode reader (Synergy LX, BioTek, Vermont, USA) and reported as the amount of Evans blue per 100 mg of dry tissue.

### Immunofluorescence staining

The paraffin sections of mice lung tissues were fixed with 4% formaldehyde, permeabilized with 0.5% Triton X-100, and then incubated with rabbit anti-CD31 antibody (#PA5-32321, Invitrogen, CA, USA,1:1000 dilution), and then incubated with secondary antibodies labeled with Alexa Fluor® Plus 594 (#A32740, Invitrogen, 1:500 dilution) and TUNEL reaction mixture (Beyotime, Shanghai, China). The nucleus was stained with DAPI. The cells were observed under fluorescence microscope, and the CD31^+^TUNEL^+^positive rate was analyzed by Image J software.

### Real-time quantitative PCR (RT-qPCR)

The expression of H19, MEG3, MALAT1, LINC-PINT, LINC00346, LINC00665, LINC00839, LINC01503, interleukin (IL-1β, IL-6, IL-18), tumor nuclear factor-α (TNF-α), caspase-1, NLRP3 were detected by RT-qPCR as previously reported [35]. Total RNA in lung tissue or cells was extracted and used for reverse transcription PCR. Then the product cDNA was used as a template for RT-qPCR. All the PCR reaction were performing in the 7500 RT-PCR system (Applied Biosystems, CA, USA). The relative expression was normalized by 2^−ΔΔCt^ method, and glyceraldehyde-3-phosphate dehydrogenase (GAPDH) was taken as the internal reference.

### Enzyme linked immunosorbent assay (ELISA)

The collected from mice and cell culture supernatant was used for ELISA. The pro-inflammatory cytokines (IL-1β, IL-6, IL-18 and TNF-α) levels were measured according to the instructions of ELISA kit (Gelatins, Shanghai, China). The OD value at 450 nm were detected by multimode reader.

### Western blot analysis

The expression of NLRP3 and cleaved caspase-1 were detected by western blot analysis as previously reported [36]. In short, total protein was extracted and quantified. 50 μg of protein sample were used for SDS-PAGE electrophoresis, and transferred to polyvinylidene fluoride membranes. Then these blots on membranes were cut (Thus, there was no blot image of adequate length), and incubated successively with the primary antibody(rabbit anti-NLRP3 antibody: #PA5-20838, rabbit anti-cleaved caspase-1 antibody: #PA5-77886, rabbit anti-GAPDH antibody: #PA1-987, Invitrogen, 1:1000 dilution)and a secondary antibody (Goat anti-Rabbit IgG, # 32460, Invitrogen, 1:500 dilution) after being blocked. Finally, the image processing system (WD-9413A, LIUYI instrument plant, Beijing, China) was used to analyze the protein expression.

### Cell viability evaluation

The cell viability was detected by MTT assay and Trypan blue staining assay. **MTT assay **[32]**:** MPVECs were incubated in 96 well plate for 24 h. Then, 20μL of pre-configured 5 mg/mL MTT solution (Sangon Biotech, Shanghai, China) was added to each well of cells, and incubated for 4 h. The absorbance at 490 nm was measured by the multimode reader. **Trypan blue staining assay **[37]**:** The adherent cells were digested with 0.5% trypsin, and made into cell suspension. After trypan blue staining, they were observed under microscope. The number of living cells in 1000 cells was counted, and the cell viability was calculated. The cell viability = the number of non-stained cells/the total number of observed cells × 100%.

### Dual-luciferase reporter gene system assay

The targeting sites in LINC00839 and miR-223 were validated by dual-luciferase reporter gene system assay as previously reported [38]. NC mimic or miR-223 mimic were co-transfected into HEK 293 T cells with pGL3 reporter plasmid and Renilla luciferase reporter plasmid containing wild-type or mutant promoter sequence of LINC00839, respectively. 24 h after transfection, the ratio of firefly and Renilla luciferase activity was measured by chemiluminescence detector (GloMax 20/20, Promega, Madison, Wisconsin, USA).

### RNA pull-down assay

The binding relationship was verified by RNA pull-down assay. First, miR-223 was labeled by Biotin probe according to the instruction of Pierce™ RNA 3′ End Desthiobiotinylation Kit (Thermo Fisher Scientific, Shanghai, China). Cell lysis was used for standby. After RNA were binding to beads, cell lysate was added to the complex, and the LINC00839 adsorbed on beads was detected by PCR.

### PI/Hoechst double staining assay

Pyroptosis in MPVECs was evaluated by PI/Hoechst double staining assay as previously reported [39]. Briefly, the cytospin specimens of MPVECs were added with Hoechst and PI solution respectively, which were incubated on ice for 30 min. The cells were observed under fluorescence microscope after washing.

### Statistical analysis

Prism 8.0 software (GraphPad software, CA, USA) was used for data analysis and graphic drawing. The experimental data were presented as “mean ± standard deviation”. The data between multiple groups were analyzed by one-way analysis of variance. The data between two groups were compared by t-test. The difference was considered statistically significant when *P* < 0.05.

## Results

### Sevoflurane reversed LPS-induced cell pyroptosis and ameliorated ALI in vivo

**Fig. 1:**
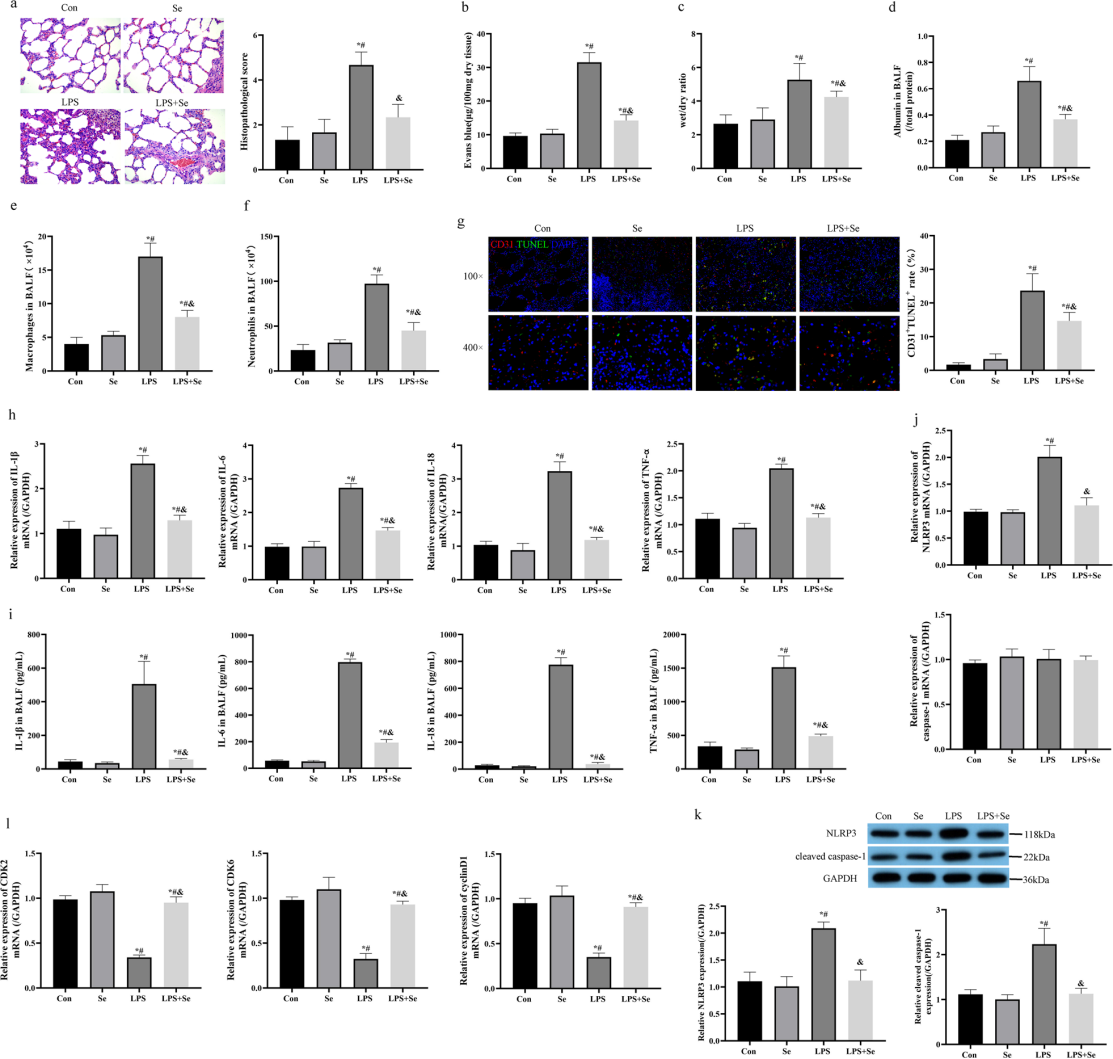
Sevoflurane reversed LPS-induced cell pyroptosis and ameliorated ALI in vivo. **a** H&E staining of mice lung tissues and the histopathological score. **b** The capillary permeability in mice lung tissue was evaluated by Evans blue staining method. **c** The wet/dry weight ratio of the lung was calculated for evaluation of pulmonary edema. **d** The albumin concentration in BALF was measured by ELISA. **e**, **f** Macrophages and neutrophils in BALF was measured by a hemocytometer. **g** Endothelial cell markers (CD31, red) and TUNEL positive cells (TUNEL, green) were observed by immunofluorescence staining. **h** The pro-inflammatory cytokines (IL-1β, IL-6, IL-18 and TNF-α) in mice lung tissues were measured by RT-qPCR. **i** The pro-inflammatory cytokines in BALF were measured by ELISA. **j**, **k** mRNA levels and protein levels of NLRP3 and cleaved caspase-1 were detected by RT-qPCR and western blot. Con, control group; Se, group treated with sevoflurane; LPS, group treated with LPS; LPS + Se, group co-treated with LPS and sevoflurane. **l** The proliferation-related factors (CDK2, CDK6, cyclinD1) in mice lung tissues were measured by RT-qPCR. *P < 0.05 compared with control group; ^#^*P* < 0.05 compared with Se group. ^&^*P* < 0.05 compared with LPS group

We established a mouse model for LPS-induced ALI as previously reported [11]. The mice lung tissues were initially examined by performing H&E staining assay. H&E results in Fig. 1a showed that LPS significantly damaged the alveolar structure: reduced the alveolar cavity and widened the alveolar wall septum, and LPS-induced super-inflammation in mice lung tissues could be apparently observed. Of note, sevoflurane partially removed LPS-induced damages in mice lung tissues and notably decreased the lung injury score (Fig. 1a). Consistently, LPS increased wet/dry ratio and promoted capillary permeability in mice lung tissues, and elevated albumin, macrophages and neutrophils in BALF, which were all counteracted by sevoflurane (Fig. 1b–f). Immunofluorescence staining results showed that LPS induced obvious death in lung endothelial cell, but sevoflurane treatment significantly decreased cell death induced by LPS (Fig. 1g). Moreover, the promoting effects of LPS on the pro-inflammatory cytokines (IL-1β, IL-6, IL-18 and TNF-α) in mice lung tissues and BALF were abolished by sevoflurane co-treatment (Fig. 1h, i). Cell pyroptosis contributes to the aggravation of LPS-induced ALI, and our data supported that LPS increased the levels of NLRP3 and cleaved caspase-1 to trigger pyroptotic cell death in mice lung tissues, which were reversed by sevoflurane (Fig. 1j, k). Meanwhile, we found that LPS suppressed proliferation-related factors (CDK2, CDK6, cyclinD1) in mice lung tissues, and sevoflurane treatment could also relief this effect (Fig. 1l). Analysis of those data illustrated that sevoflurane ameliorated LPS-induced detrimental symptoms in mice lung tissues.

### Sevoflurane suppressed LPS-induced pyroptotic cell death in MPVECs in vitro

**Fig. 2:**
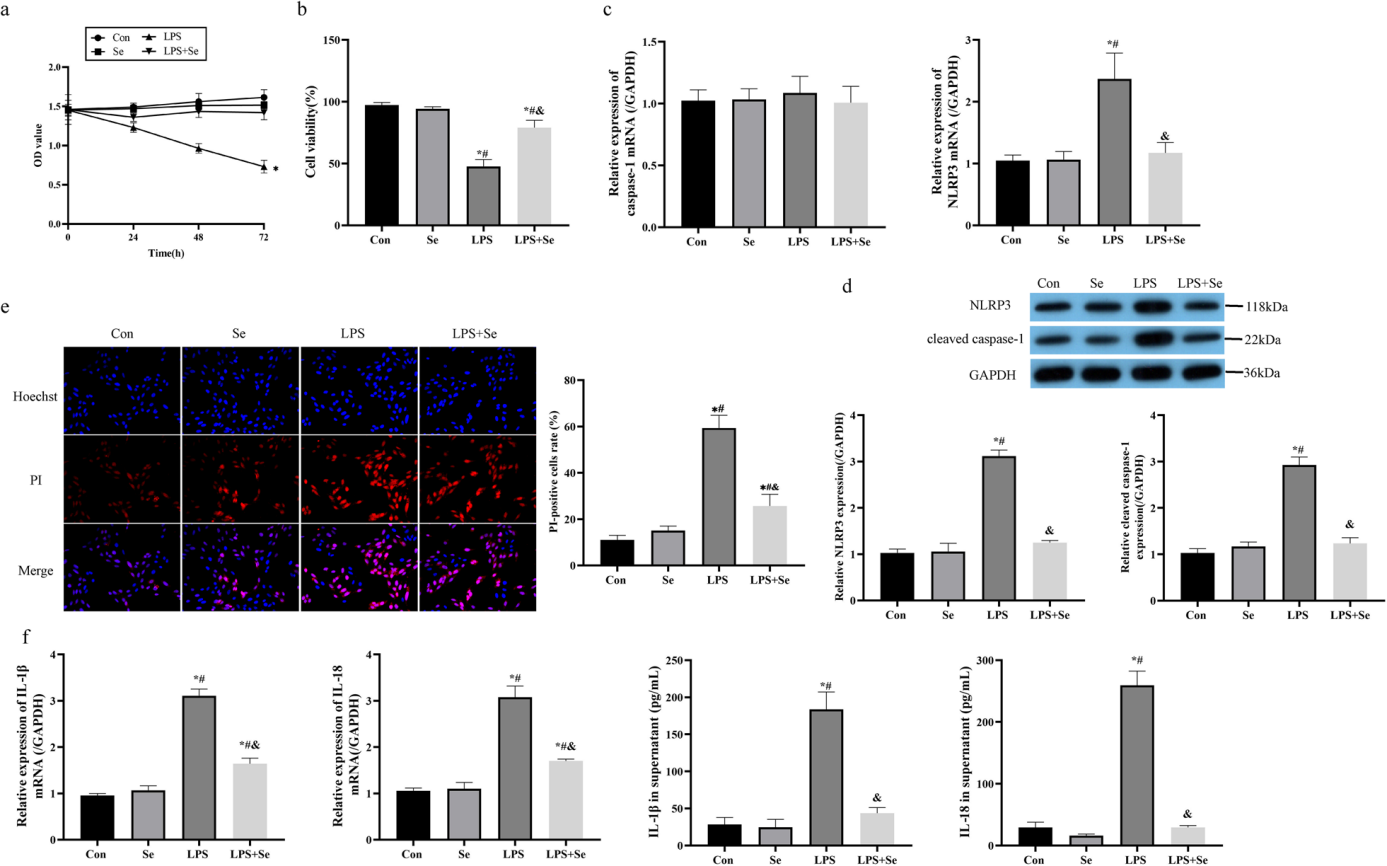
Sevoflurane suppressed LPS-induced pyroptotic cell death in MPVECs in vitro. The MPVECs were subjected to LPS treatment, and then intervened with sevoflurane. **a**, **b** The cell viability was detected by the MTT assay (**a**) and trypan blue staining assay (**b**). **c** The mRNA levels of NLRP3 and caspase-1 were detected by RT-qPCR analysis. **d** The protein levels of NLRP3 and cleaved caspase-1 were detected by western blot analysis. **e** The pyroptotic cells were detected by PI/Hoechst double staining assay. (f) The pro-inflammatory cytokines (IL-1β, and IL-18) in MPVECS and cell supernatant were measured by RT-qPCR and ELISA. Con, control group; Se, group treated with sevoflurane; LPS, group treated with LPS; LPS + Se, group co-treated with LPS and sevoflurane. *P < 0.05 compared with control group; ^#^*P* < 0.05 compared with Se group. ^&^*P* < 0.05 compared with LPS group

The MPVECs were subjected to LPS treatment in vitro to mimic the realistic conditions of LPS-induced ALI progression in vivo. Then, the MTT assay and trypan blue staining assay results evidenced that LPS suppressed cell viability in MPVECs in a time-dependent manner, and the viability of those LPS-treated MPVECs was rescued by sevoflurane (Fig. 2a, b). In addition, the RT-qPCR and Western Blot analysis evidenced that the expressions of NLRP3 and cleaved caspase-1 were elevated by LPS in MPVECs, and the LPS-induced upregulation of the pyroptosis signatures were abrogated by sevoflurane (Fig. 2c, d). Also, the cells were synergistically stained with PI and Hoechst, and the data supported that LPS-induced increase of the ratio of PI-positive cells were abolished by sevoflurane (Fig. 2e). Further data evidenced that sevoflurane restrained the generation and secretion of IL-1β and IL-18 in the LPS-treated MPVECs and its supernatants (Fig. 2f). Those data convinced us that sevoflurane ameliorated LPS-induced pyroptotic cell death in MPVECs in vitro.

### Sevoflurane suppressed LINC00839 to recover cellular functions in LPS-treated MPVECs

**Fig. 3:**
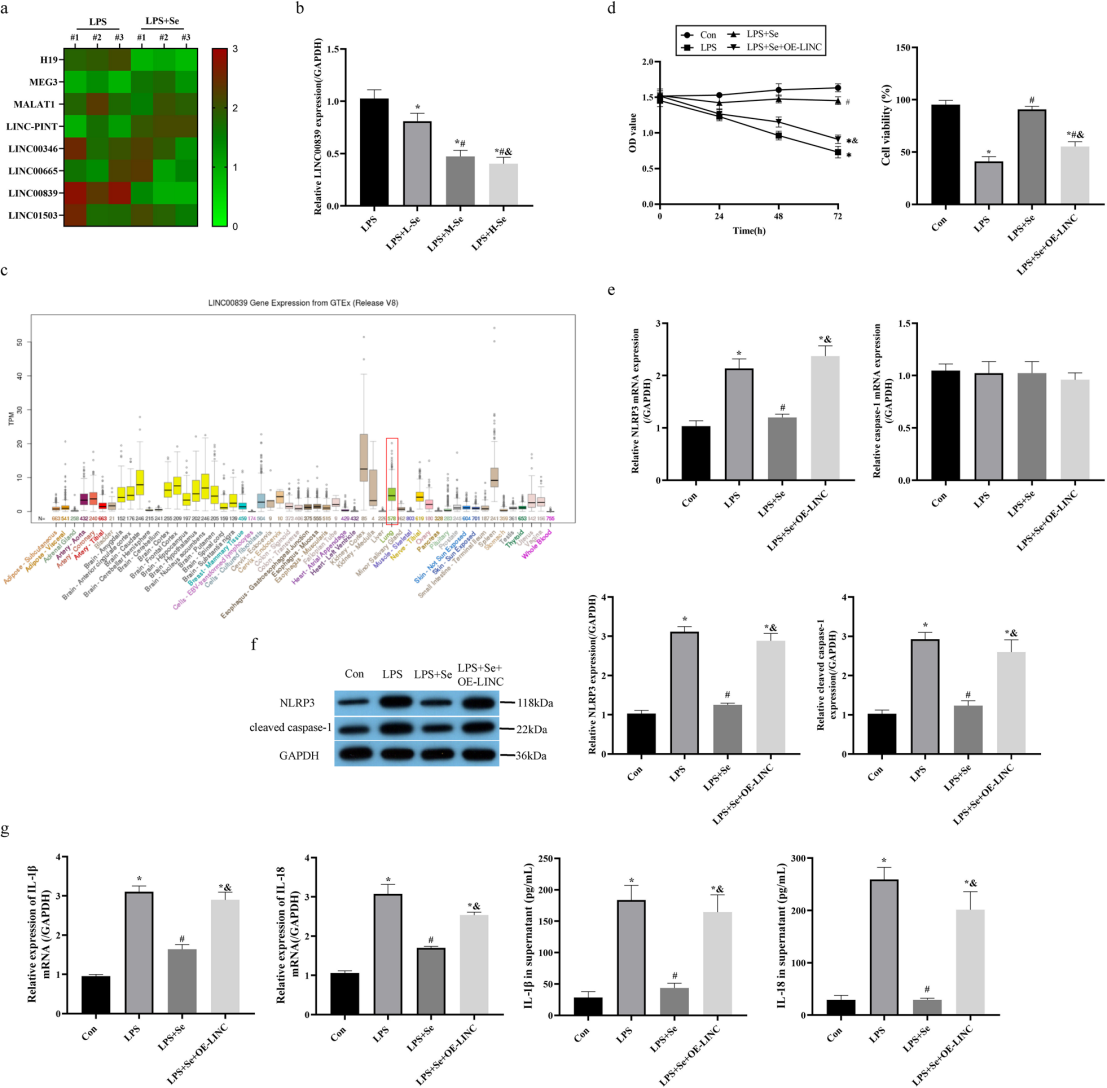
Sevoflurane suppressed LINC00839 to recover cellular functions in LPS-treated MPVECs. **a** The proliferation-associated LncRNAs in ALI model mice treated with or without sevoflurane were screened by RT-qPCR, and analyzed by heat map. **b** The LINC00839 expressions were detected in MPVECs treated with different concentration of sevoflurane, L-Se: 1% of sevoflurane, M-Se:3% of sevoflurane, L-Se:5% of sevoflurane. **c** The distribution of LINC00839 in the organization were inferred from the public database of GTEx project, and the expression in lung tissue was marked with a red box. MPVECs were treated with overexpression of LINC00839 on the basis of the treatment of LPS and sevoflurane: **d** The cell viability was determined by MTT assay and trypan blue staining assay. **e** The mRNA levels of NLRP3 and caspase-1 were detected by RT-qPCR analysis. **f** The protein levels of NLRP3 and cleaved caspase-1 were detected by western blot analysis. **g** The levels of IL-1β, and IL-18 in MPVECS and cell supernatant were measured by RT-qPCR and ELISA. Con, control group; LPS, group treated with LPS; LPS + Se, group co-treated with LPS and sevoflurane. LPS + Se + OE-LINC, group treated with overexpression of LINC00839 on the basis of the treatment of LPS and sevoflurane. *P < 0.05 compared with control group; ^#^*P* < 0.05 compared with LPS group. ^&^*P* < 0.05 compared with LPS + Se group

Given that sevoflurane is reported to regulate cellular functions through various LncRNAs [40–42], we performed RT-qPCR to screen the possible LncRNAs that involved in the process of sevoflurane-mediated protective effects in LPS-induced ALI. As shown in Fig. 3a, the proliferation-associated LncRNAs were screened by RT-qPCR, and we noticed that LINC00839 was significantly downregulated by sevoflurane in ALI mice tissues. Further cellular experiments validated that sevoflurane also suppressed LINC00839 expressions in LPS-treated MPVECs in a dose dependent manner (Fig. 3b). Thus, we selected LINC00839 for further analysis. It was inferred from the public database of Genotype Tissue Expression (GTEx) project (http://genome.ucsc.edu/gtexBodyMap.html) that LINC00839 is widely distributed in different organs of human, including lung tissues (Fig. 3c). Next, LINC00839 was overexpressed in MPVECs. The data showed that upregulation of LINC00839 apparently suppressed cell viability in the MPVECs co-treated with sevoflurane and LPS (Fig. 3d). The following experiments evidenced that overexpression of LINC00839 abolished the suppressing effects of sevoflurane on NLRP3 and cleaved caspase-1 expressions in the LPS-treated MPVECs (Fig. 3e, f). Further results also supported that sevoflurane restrained LPS-induced generation/secretion of IL-1β and IL-18 in vitro via downregulating LINC00839 (Fig. 3g). Collectively, summary of the data hinted that sevoflurane exerted its anti-pyroptotic effects in LPS-treated MPVECs through modulating LINC00839.

### Silencing of LINC00839 restrained LPS-induced cell pyroptosis in MPVECs

**Fig. 4:**
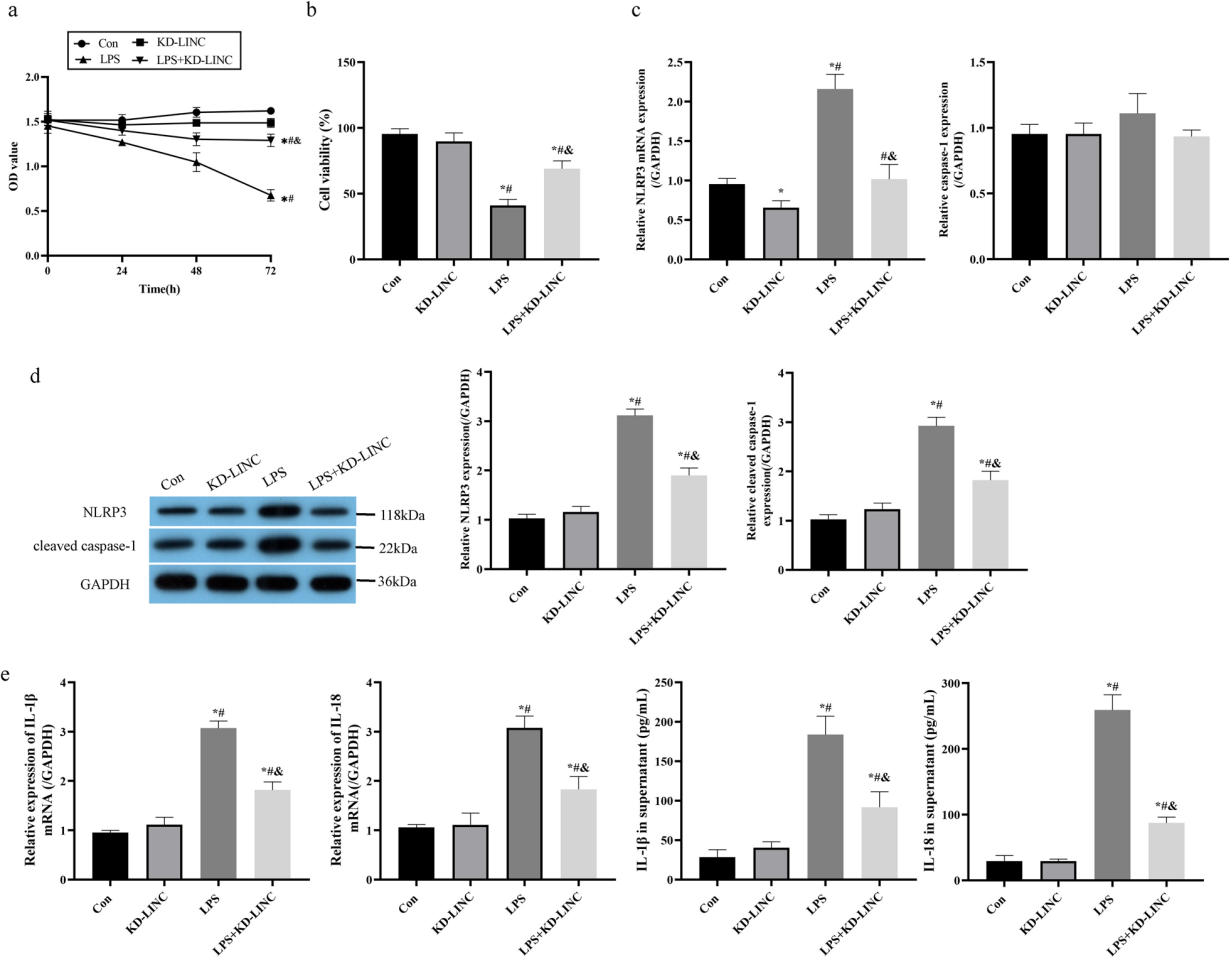
Silencing of LINC00839 restrained LPS-induced cell pyroptosis in MPVECs. LINC00839 was silenced in the LPS-treated MPVECs. **a**, **b** The cell viability was determined by MTT assay (**a**) and trypan blue staining assay (**b**). **c** The mRNA levels of NLRP3 and caspase-1 were detected by RT-qPCR analysis. **d** The protein levels of NLRP3 and cleaved caspase-1 were detected by western blot analysis. **e** The levels of IL-1β, and IL-18 in MPVECS and cell supernatant were measured by RT-qPCR and ELISA. Con, control group; KD-LINC, group with LINC00839 knockdown; LPS, group treated with LPS; LPS + KD-LINC, group with knockdown of LINC00839 on the basis of LPS treatment. *P < 0.05 compared with control group; ^#^*P* < 0.05 compared with KD-LINC group. ^&^*P* < 0.05 compared with LPS group

The biological functions of LINC00839 itself on regulating LPS-induced cell pyroptosis and viability in MPVECs were further discussed, and LINC00839 was silenced in the LPS-treated MPVECs. The results in Fig. 4a, b suggested that the inhibiting effects of LPS on cell viability in MPVECs were significantly rescued by LINC00839 ablation. Also, as determined by RT-qPCR and Western Blot analysis, we showed that knockdown of LINC00839 decreased the expression levels of NLRP3 and cleaved caspase-1 in LPS-treated MPVECs (Fig. 4c, d). Furthermore, the promoting effects of LPS on IL-1β and IL-18 expressions were abolished by silencing LINC00839 (Fig. 4e), suggesting that knockdown of LINC00839 restored cell viability and restrained cell pyroptosis in LPS-treated MPVECs.

### LINC00839 regulated NLRP3/cleaved caspase-1 pathway through miR-223

**Fig. 5:**
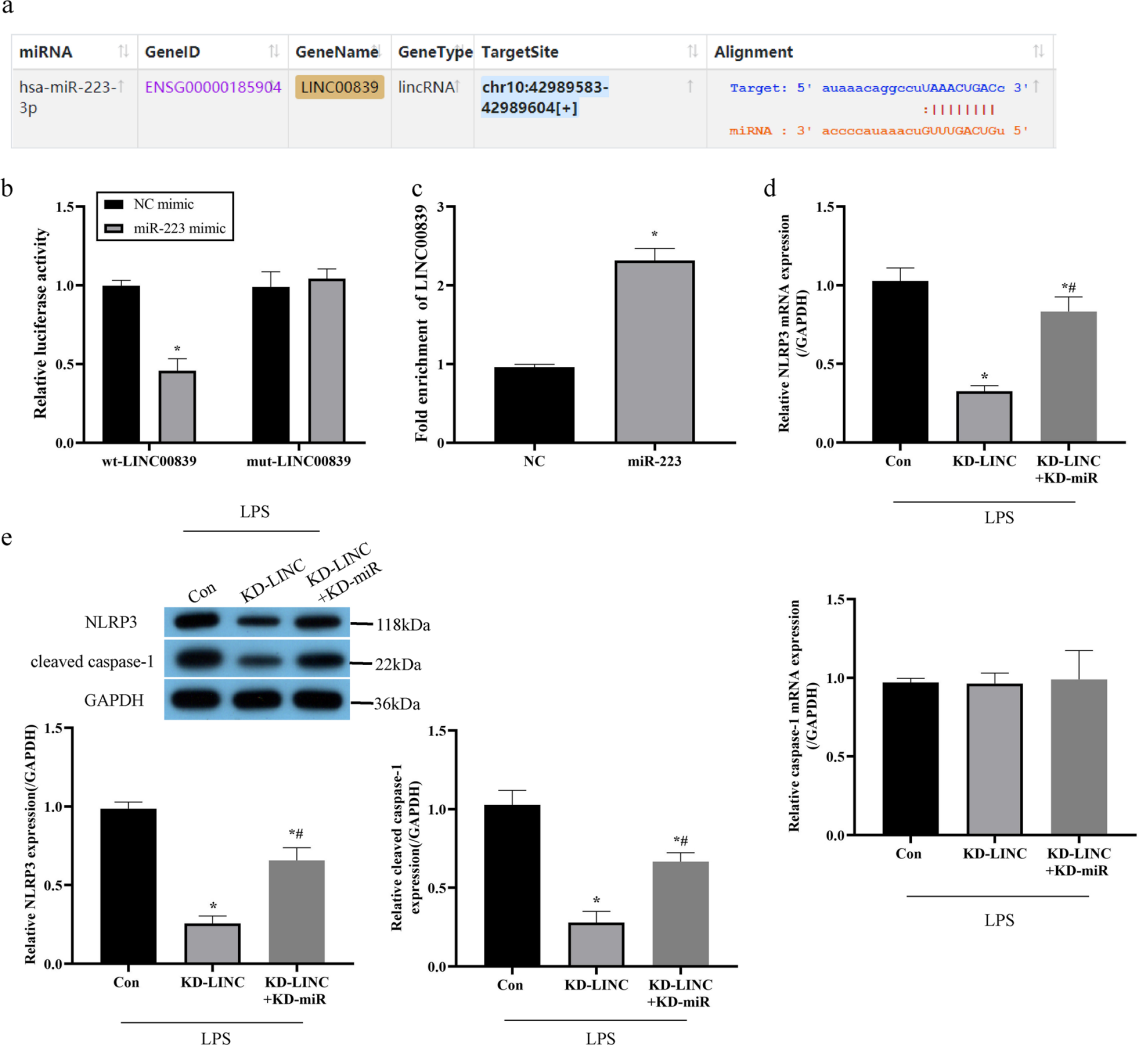
LINC00839 regulated NLRP3/cleaved caspase-1 pathway through miR-223. **a** The target relationship between LINC00839 and miR-223 was predicted on the starbase website. **b** The targeting sites in LINC00839 and miR-223 were validated by dual-luciferase reporter gene system assay. **c** The binding relationship was verified by RNA pull-down assay. LINC00839 and miR-223 were silenced in MPVECS: **d** The mRNA expression of NLRP3 and caspase-1 was measured by RT-qPCR. **e** The protein levels of NLRP3 and cleaved caspase-1 were detected by western blot analysis. Wt, wild-type; mut, mutant. Con, control group; KD-LINC, group with LINC00839 knockdown; KD-LINC + KD-miR, group with knockdown of LINC00839 and miR-223. *P < 0.05 compared with control group; ^#^*P* < 0.05 compared with KD-LINC group

Given that the regulatory effects between LINC00839 and NLRP3 have been described, the classic ceRNA network mechanisms encouraged us to hypothesize that there might exist miRNAs that linked LINC00839 and NLRP3 in the present study. Through the StarBase website (https://starbase.sysu.edu.cn/agoClipRNA.php?source=mRNA), we predicted that the sequences located in the chr10:42989583–42989604[+] of LINC00839 were capable of binding to miR-223(Fig. 5a), which is verified as a pivotal upstream regulator for NLRP3 by targeting its 3’ untranslated regions [17, 28, 29]. The targeting sites in LINC00839 and miR-223 were validated by performing the dual-luciferase reporter gene system assay, which found that miR-223 mimic especially suppressed luciferase activities in the MPVECs co-transfecting with wild-type LINC00839, instead of the LINC00839 vectors with mutated binding sequences (Fig. 5b). Further RNA pull-down assay verified that LINC00839-probes were able to pull down miR-223 (Fig. 5c). Next, according to the published data that miR-223 targeted 3’ untranslated region of NLRP3 for its degradation [17, 28, 29], we evidenced that LINC00839 positively regulated NLRP3 in a miR-223-depednent manner. Specifically, the suppressing effects of LINC00839 knockdown on NLRP3 expressions in LPS-treated MPVECs were reversed by knockdown of miR-223 (Fig. 5d, e). Taken together those data, we summarized that silencing of LINC00839 abolished LPS-induced NLRP3 upregulation by releasing miR-223.

### Sevoflurane attenuated LPS-induced cell death and inflammation in MPVECs via the miR-223/NLRP3 axis

**Fig. 6:**
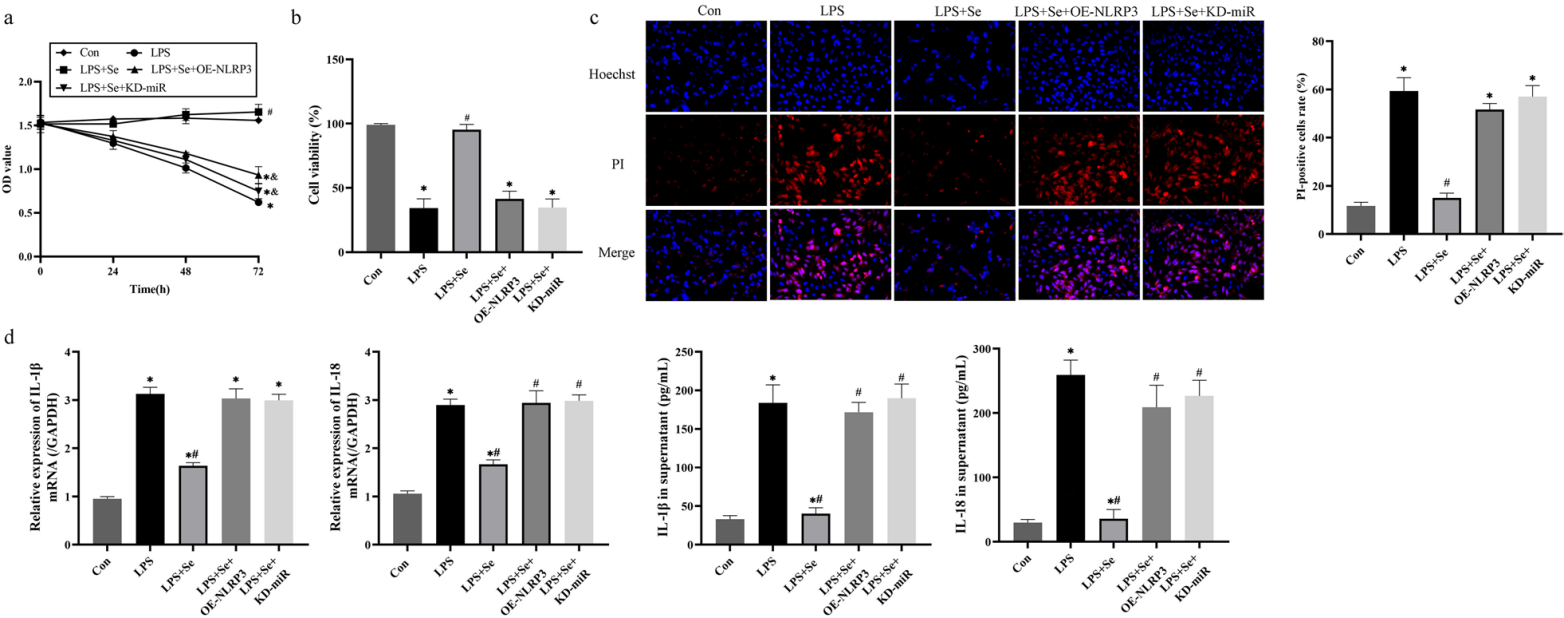
Sevoflurane attenuated LPS-induced cell death and inflammation in MPVECs via the miR-223/NLRP3 axis. The MPVECs were treated with overexpression of NLRP3 or knockdown of miR-223 on the basis of LPS and sevoflurane treatment. **a**, **b** The cell viability was detected by the MTT assay (**a**) and trypan blue staining assay (**b**). **c** The pyroptotic cells were detected by PI/Hoechst double staining assay. **d** The levels of IL-1β, and IL-18 in MPVECS and cell supernatant were measured by RT-qPCR and ELISA. LPS, group treated with LPS; LPS + Se, group co-treated with LPS and sevoflurane. LPS + Se + OE-NLRP3, group with overexpression of NLRP3 on the basis of LPS and sevoflurane treatment. LPS + Se + KD-miR, group with knockdown of miR-223 on the basis of LPS and sevoflurane treatment. *P < 0.05 compared with LPS group; ^#^*P* < 0.05 compared with LPS + Se group

Finally, we investigated whether sevoflurane ameliorated LPS-induced cytotoxicity in MPVECS through modulating the miR-223/NLRP3 axis. Therefore, miR-223 was silenced, whereas NLRP3 was overexpressed in MPVECs. The results showed that both miR-223 ablation and NLRP3 overexpression abrogated the protective effects of sevoflurane on LPS-treated MPVECs regarding to cell viability (Fig. 6a, b). Further results supported that sevoflurane decreased the ratio of PI-positive pyroptotic cells in the MPVECs treated with LPS, which were reversed by miR-223 ablation and NLRP3 overexpression (Fig. 6c). Consistently, silencing of miR-223 and upregulation of NLRP3 were capable of increasing IL-1β and IL-18 expression levels to abolish the anti-inflammatory effects of sevoflurane on LPS-treated MPVECs (Fig. 6d). Thus, we drawn the conclusions from those data that sevoflurane exerted its protective effects on LPS-induced cytotoxicity in MPVECs via modulating the miR-223/NLRP3 axis.

## Discussion

**Fig. 7:**
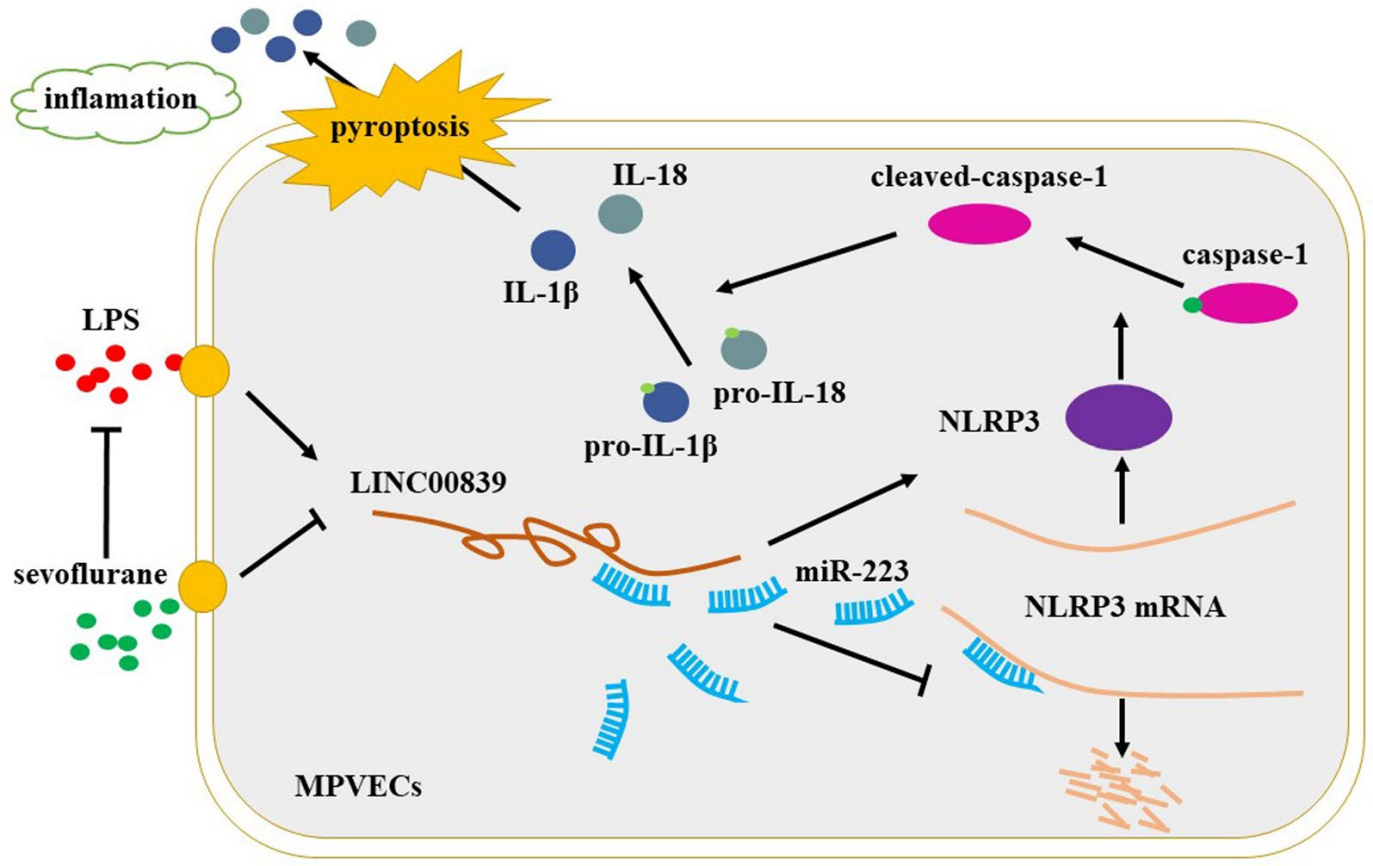
Sevoflurane ameliorates LPS-induced ALI by modulating the LncRNA LINC00839/miR-223/NLRP3 axis. Sevoflurane diminishes LPS-induced upregulation of LINC00839/NLRP3, leads to inactivation of caspase-1 and reduction of pro-inflammatory factor (IL-1β and IL-18), and suppresses pyroptosis and inflammation, thereby ameliorating LPS-induced ALI

Our present work revealed the detailed molecular mechanism of sevoflurane in ALI. We hypothesized that sevoflurane might protect lung tissues through downregulating LINC00839/miR-223/NLRP3 axis. Firstly, we verified sevoflurane ameliorated LPS-induced detrimental symptoms of ALI in vivo and LPS-induced pyroptotic cell death in vitro. Subsequently, we identified that sevoflurane downregulated LINC00839 to ameliorate LPS-induced ALI. Finally, we validated that LINC00839 acts as a ceRNA to upregulate NLRP3 mRNA via sequestering miR-223, and the LINC00839/miR-233 axis was the regulatory target of sevoflurane in ALI (Fig. 7).

ALI or its now unified term, acute respiratory distress syndrome (ARDS), has been a major cause of death in intensive care unit [43, 44]. Despite the tremendous efforts of the medical community, the mortality rate of ARDS is still high [45, 46]. It is necessary to further investigate the new targets of ARDS, in order to better improve the management of ARDS. Previous studies have shown that sevoflurane can provide lung protect effect in LPS-induced ALI by inhibiting inflammation [11, 31, 47].Of note, a clinical study by Jabaudon et al. [48] verified that sevoflurane improves oxygenation, and alleviates epithelial injury and inflammation in patients with ARDS. This study provides the first clinical evidence of sevoflurane's protective effect on lung tissue. However, the molecular mechanism of sevoflurane in lung protection is largely unknown. LncRNAs participates in almost all physiological and pathological processes. LINC00839, a novel LncRNA, is originally regarded as a cancer-promoting gene [23, 25, 49]. It’s the first study to confirm the involvement of LINC00839 in the ALI induced by LPS.

In this study, we confirmed that LINC00839, acts as the upstream of the miR-223/NLRP3 axis to play its role in ALI. It is another brand-new discovery. The targeting interaction between miR-223 and NLRP3 in inflammatory diseases has been reported by numerous studies [26, 50–52]. One study by Zhang et al. [53] found that LncRNA MEG3 acts as an endogenous sponge to inhibit the function of miR-223 and increase NLRP3 expression. A study by Ji et al. [17] suggested LncRNA OIP5-AS1 exacerbates LPS-induced ALI also through miR-223/NLRP3 axis. Another study by Yan et al. [29] showed that miR-223 over expression reduces NLRP3 mediated inflammation in ALI model. All these studies demonstrated the miR-223/NLRP3 axis mediates inflammation and pyroptosis in ALI. Differently, the present finding revealed its upstream regulatory molecule of sevoflurane in ALI.

Pyroptosis, also known as inflammatory necrosis, is characterized by the activation of a strong inflammatory response [16, 17]. As a result, pyroptosis is considered to be a key player in the progression of ALI [54, 55]. Activation of NLRP3 inflammasome plays a critical role in inflammation and pyroptosis during ALI [56]. After NLRP3 inflammasome activation, caspase-1 is cleaved and activated, thereby promoting the maturation and release of pro-inflammatory cytokines, such as IL-1β and IL-18 [57, 58], as displayed in Fig. 7. As it progresses, the inflammation is constantly amplified and the cells appear pyronecrotic necrosis, finally aggravating the lung tissue injury [18]. The present study verified that sevoflurane could reverse LPS-induced inflammation and pyroptosis through LINC00839/miR-223/NLRP3 axis.

Notably, the term ALI, which was formerly signified as a mild form of ARDS, has been clinically replaced by ARDS since the Berlin consensus conference. In the present study, we mainly used the LPS-induced ALI model to test the molecular mechanism, so the term ALI was still adopted for description here. Therefore, our results provided more evidences for the benefit of sevoflurane in the prevention of ARDS.

## Conclusions

Taken together, our results show that sevoflurane ameliorates inflammation and pyroptosis in LPS-induced ALI via the novel LINC00839/miR-223/NLRP3 axis. The finding improves the understanding of sevoflurane’s lung-protective effect in ARDS, and might provide a potential target for the prevention of ARDS.

## Data Availability

All data generated or analyzed during this study are included in this published article and the supplementary original blots.

## Abbreviations

ALI: Acute lung injury
LPS: Lipopolysaccharide
MPVECs: Mouse pulmonary microvascular endothelial cells
LncRNA: Long non-coding RNA
ceRNA: Competing endogenous RNA
BALF: Bronchoalveolar lavage fluid
RT-qPCR: Real-Time quantitative PCR
IL-1β: Interleukin-1β
IL-6: Interleukin-6
IL-18: Interleukin-18
TNF-α: Tumor nuclear factor-α
H&E: Hematoxylin & Eosin
ELISA: Enzyme linked immunosorbent assay
NLRP3: NOD-like receptor protein 3
GAPDH: Glyceraldehyde-3-phosphate dehydrogenase
ARDS: Acute respiratory distress syndrome
